# Autophagy in Neutrophils: From Granulopoiesis to Neutrophil Extracellular Traps

**DOI:** 10.3389/fcell.2018.00109

**Published:** 2018-09-04

**Authors:** Panagiotis Skendros, Ioannis Mitroulis, Konstantinos Ritis

**Affiliations:** ^1^Laboratory of Molecular Hematology, Department of Medicine, Democritus University of Thrace, Alexandroupolis, Greece; ^2^First Department of Internal Medicine, University Hospital of Alexandroupolis, Democritus University of Thrace, Alexandroupolis, Greece; ^3^Institute for Clinical Chemistry and Laboratory Medicine, Technische Universität Dresden, Dresden, Germany; ^4^National Center for Tumor Diseases, Dresden, Germany

**Keywords:** autophagy, neutrophil, granulopoiesis, phagocytosis, degranulation, neutrophil extracellular traps, inflammation

## Abstract

Autophagy is an evolutionarily conserved intracellular degradation system aiming to maintain cell homeostasis in response to cellular stress. At physiological states, basal or constitutive level of autophagy activity is usually low; however, it is markedly up-regulated in response to oxidative stress, nutrient starvation, and various immunological stimuli including pathogens. Many studies over the last years have indicated the implication of autophagy in a plethora of cell populations and functions. In this review, we focus on the role of autophagy in the biology of neutrophils. Early studies provided a link between autophagy and neutrophil cell death, a process essential for resolution of inflammation. Since then, several lines of evidence both in the human system and in murine models propose a critical role for autophagy in neutrophil-driven inflammation and defense against pathogens. Autophagy is essential for major neutrophil functions, including degranulation, reactive oxygen species production, and release of neutrophil extracellular traps. Going back to neutrophil generation in the bone marrow, autophagy plays a critical role in myelopoiesis, driving the differentiation of progenitor cells of the myeloid lineage toward neutrophils. Taken together, in this review we discuss the functional role of autophagy in neutrophils throughout their life, from their production in the bone marrow to inflammatory responses and NETotic cell death.

## Introduction

Macroautophagy (hereafter called autophagy) is an intracellular homeostatic mechanism of eukaryotic cells, which is essential for the cellular response to starvation and other types of cell stress including hypoxia, oxidative burst, DNA damage, and infection (Levine et al., 2011). During autophagy, cytosolic constituents are enclosed in double-membrane vesicles, called autophagosomes, and subsequently delivered to lysosomes for degradation (autolysosomes). This dynamic, tightly regulated, biological process protects cell by sensing and clearing damaged cellular elements or intracellular pathogens, providing nutrient supply through recycling of cytosolic macromolecules and organelles (Sil et al., 2018).

Over the last years, a large body of evidence implicates autophagy in several host immune functions, such as phagocytosis, elimination of intracellular pathogens, antigen presentation, thymic selection, maintenance of lymphocyte homeostasis, and regulation of cytokine production (Skendros and Mitroulis, 2012; Deretic et al., 2015; Sil et al., 2018). On the other hand, aberrant or uncontrolled autophagy may lead to autophagy-dependent cell death (Galluzzi et al., 2018). Thus, autophagy is implicated in both cell survival and death, depending on the cell type and stress conditions. Dysregulated autophagy has been associated with a wide range of diseases, including inflammatory diseases, neurodegenerative disorders, and cancer (Levine et al., 2011; Galluzzi et al., 2015; Menzies et al., 2015; Martinez et al., 2016).

Neutrophils represent the most abundant effector cells of immune system in humans and are the first to migrate from bloodstream to sites of tissue inflammation, in response to invading pathogens or host-derived mediators (Mayadas et al., 2014). They are short-lived cells, with a circulating half-life varying from 6–8 h to few days. At steady-state conditions renewal of neutrophils is ensured by constant bone marrow granulopoiesis (Cowland and Borregaard, 2016). However, during severe, systemic, inflammatory settings, a reprogramming of haematopoietic response is commenced, leading to *de novo* generation of high numbers of neutrophils from myeloid progenitors and their mobilization to circulation, in a process called emergency granulopoiesis (Manz and Boettcher, 2014).

Currently, the traditional concept that neutrophils comprise terminally differentiated cells with limited plasticity and highly conserved function, due to their low transcriptional activity, has been revised. Neutrophils express a wide variety of surface receptors that gives them the ability to respond quickly according to disease environmental cues and undergo transcriptional reprogramming leading to *de novo* synthesis of cytokines (Tamassia et al., 2018). This adaption makes neutrophils a phenotypically and functionally heterogeneous cell population (Scapini et al., 2016; Silvestre-Roig et al., 2016; Jablonska and Granot, 2017). Accordingly, upon activation, neutrophils are able to exert their antimicrobial and pro-inflammatory functions by using three distinct mechanisms: phagocytosis, degranulation and the most recently described formation and release of neutrophil extracellular traps (NETs) (Mayadas et al., 2014; Mitsios et al., 2016).

The first evidence that autophagy occurs in human neutrophils provided by Mitroulis et al. (2010) indicated the induction of autophagy machinery in a both phagocytosis-dependent (*Escherichia coli*) and phagocytosis-independent manner (such as IL-1β, TLR agonists, rapamycin, and PMA) (Mitroulis et al., 2010). Since then, many studies have indicated that autophagy is crucially involved in neutrophil biology and its effector functions. This review summarizes the biological role of autophagy in the regulation of granulopoiesis and neutrophil/NETs-driven antimicrobial defense and inflammation, by discussing recent evidence derived from experimental and clinical studies, as well as, potential, autophagy-based, therapeutic strategies against neutrophil-mediated diseases.

## Essentials of the Molecular Machinery of Autophagy

The term autophagy was first introduced by Christian de Duve in 1963 to characterize the ability of lysosomes in self-eating (Levine and Klionsky, 2017). In 1990s, Y. Ohsumi and co-workers identified in yeast the genes that govern the autophagy-related pathways and showed that they are conserved from yeast to mammalian cells, paving the way for the study of autophagy in human health and disease (Takeshige et al., 1992; Tsukada and Ohsumi, 1993; Mizushima et al., 1998).

Today, the functional complexes of autophagy-related (ATG) proteins and many of the molecular events that underline the sequential steps of autophagy, from the initiation of autophagosome formation to fusion with lysosome and formation of autolysosome, have been extensively investigated and well described (Yu et al., 2018). In brief, upon autophagy-related stimuli ATG proteins are activated and recruited to begin the formation of autophagosome as an isolation membrane (phagophore), deriving from rough endoplasmic reticulum subdomain, the omegasome (Levine et al., 2011; Hurley and Young, 2017). In mammals, autophagosome initiation is executed in cooperation with two cardinal protein complexes: the serine threonine kinase complex unc51-like autophagy activating kinase 1 (ULK1) composed of ULK1, ATG13, FAK family kinase-interacting protein (FIP)200 and ATG101, and the downstream class III PI 3-kinase complex I (PI3KC3–C1) that includes PI3KC3/VPS34, PIK3R4/VPS15, Beclin 1, ATG14, and nuclear receptor binding factor 2 (NRBF2) (Itakura et al., 2008; Backer, 2016; Lin and Hurley, 2016). Consequently, PI3KC3–C1 catalyzes the production of phosphatidylinositol 3-phosphate (PI3P), leading to the recruitment of two key ubiquitin-like conjugation systems, the ATG5-12 and the microtubule-associated protein 1 light chain 3 beta (LC3B). ATG5-12 system induces the LC3B I lipidation to generate LC3B II. LC3B-II lipidated protein is translocated at nascent autophagosomal membrane and facilitates growth, elongation, and curvature of the forming autophagosomes (Ichimura et al., 2000; Wild et al., 2014).

Maturation of autophagosome to autolysosome is the final, degradative, step of autophagic molecular machinery. Autophagosomes loose the inner of the two membranes upon fusion with lysosomes to form autolysosomes, assigned to degrade sequestered cytoplasmic cargo by hydrolases. Autophagosome maturation is directed by the molecular complex hVPS34-Beclin 1 in association with hVPS38 (UVRAG) (Liang et al., 2008; Backer, 2016).

Induction of autophagy machinery is regulated by the energy sensing system of AMP-activated kinase (AMPK)/mechanistic target of rapamycin (mTOR) complex 1 (mTORC1) (Laplante and Sabatini, 2012; Sil et al., 2018). During nutrient/energy starvation ATP levels decrease and AMPK is activated, whereas mTORC1 is inactivated. AMPK promotes autophagy by directly activating the pre-initiation complex ULK1 through phosphorylation, and inhibition of mTORC1 permits activated ULK1 complex translocation at early autophagosomal structures to exert its inductive effect (Egan et al., 2011; Kim et al., 2011). Therefore, inactivation of mTORC1 is a major trigger for autophagy (Kim and Guan, 2015; Sil et al., 2018).

## Autophagy and Regulation of Granulopoiesis

Granulopoiesis, i.e., the generation of granulocytes at steady state conditions or upon hematopoietic stress, including myeloablation or systemic inflammation, is a tightly regulated cascade of events that involves not only committed precursors of this specific lineage, but also hematopoietic stem and progenitor cell (Mitroulis et al., 2018a). Several lines of evidence suggest that cellular metabolism is critical in the regulation of the balance between maintenance of hematopoietic stem cells (HSC) and lineage differentiation (Suda et al., 2011; Mitroulis et al., 2018b). HSCs depend on glycolysis to meet their needs for energy production in the highly hypoxic bone marrow microenvironment (Suda et al., 2011; Takubo et al., 2013; Wang et al., 2014). A switch in their metabolic status from glycolysis to mitochondrial metabolism and oxidative phosphorylation (OXPHOS) has been shown to result in the loss of stemness and the differentiation of hematopoietic progenitors, mainly due to the production of intracellular reactive oxygen species (ROS) (Suda et al., 2011; Maryanovich et al., 2015). Fatty acid oxidation (FAO) has been also shown to negatively regulate HSC maintenance and result in their differentiation to multipotent and lineage committed progenitor cells (Ito et al., 2012).

**FIGURE 1:**
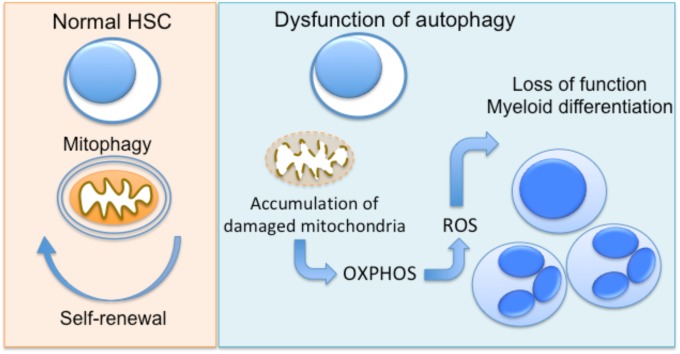
Autophagy regulates hematopoietic progenitor function. In normal hematopoietic stem cells (HSC), autophagy enables the clearance of damaged mitochondria (mitophagy), promoting the maintenance of HSC function. Dysfunctional autophagy, due to deficiency of autophagy genes *Atg7* or *Atg12* in mice has been linked to accumulation of mitochondria and metabolic reprogramming toward OXPHOS. This results in functional decline and differentiation toward myeloid lineage.

Several lines of evidence propose an important role for autophagy as a regulator of cellular metabolism in HSC, and as a result, in the regulation of quiescence and differentiation of these cells (Kohli and Passegué, 2014; García-Prat et al., 2017). The clearance of damaged mitochondria by mitophagy prevents the accumulation of ROS in HSCs, which leads to their damage and, finally, apoptosis (Joshi and Kundu, 2013). Loss of *Atg7* in HSC results in impaired HSC function, probably due to the accumulation of damaged mitochondria and production of ROS (Mortensen et al., 2011). The same group also demonstrated that hematopoietic deletion of *Atg7* resulted in robust myeloproliferation with features resembling acute myeloid leukemia (Mortensen et al., 2011). An other study by Warr et al. (2013) demonstrated that forkhead box O3 (FOXO3A)-mediated induction of autophagy is protective for HSC, enabling their survival upon metabolic stress. The homeostatic role of autophagy in hematopoietic progenitor function is also supported by a recent study showing that hyperactive mitophagy due to deletion of the gene encoding the AAA+-ATPase Atad3a has a detrimental effect on HSCs homeostasis, skewing differentiation to myeloid lineage (Jin et al., 2018). Using several genetic mouse models, Ho et al. (2017) further demonstrated that the critical interplay between autophagy and cell metabolism in HSCs leads to epigenetic changes and loss of stemness. Disruption of autophagy due to *Atg12* deficiency resulted in metabolic reprogramming of HSC toward OXPHOS and myeloid lineage bias, resembling the phenotype of activated HSC (Ho et al., 2017). Interestingly, autophagic activity in a subset of HSC during aging is linked with protection against the expected with functional decline of hematopoietic progenitors (Ho et al., 2017), being in line with the well-established role of autophagy in the maintenance of cellular health (He and Klionsky, 2009). Taken together, autophagy has a major involvement in the regulation of early progenitors of hematopoietic system (**Figure 1**).

Even though the contribution of autophagy in HSCs is well established, its role in the progression of later stages of progenitors of myeloid lineage is less studied. Myeloid cell specific deletion of *Atg5* has been shown to positively regulate the proliferation rate of neutrophil precursors without being essential for granulopoiesis, leading to accumulation of neutrophils in the bone marrow, blood and spleen, without affecting the functionality of neutrophils in terms of effector functions, apoptosis and migration (Rožman et al., 2015). A seminal study by Riffelmacher et al. (2017) further reinforced the importance of autophagy in granulopoiesis. In this study, it was shown that the degradation of lipid droplets by autophagy is necessary to fuel OXPHOS with fatty acids, resulting in a swift from glycolysis to OXPHOS, a process necessary for the late stages of neutrophil differentiation (Riffelmacher et al., 2017). Finally, a recent study by Huang et al. (2018) reported the differential expression of 22 autophagy-related genes between monocytic and granulocytic differentiation, proposing a role for autophagy in the late stages of differentiation of myeloid precursors toward granulocytes and monocytes.

## Interaction Between Autophagy and Phagocytosis

Phagocytosis and production of ROS are pivotal mechanisms of microbial killing in neutrophils. Several studies have indicated the dynamic interplay between autophagy and phagocytosis in host defense in macrophages (Sanjuan et al., 2007; Gong et al., 2011; Martinez et al., 2015). Autophagy is able to detect and eliminate intracellular pathogens that escape from endocytic compartments of phagocytosis. Pattern recognition receptors (PRRs), such as Toll-like receptors (TLRs), nucleotide-binding oligomerization domain-containing protein (NOD)1/2, and the ubiquitin-binding protein p62/SQSTM1 become activated by sensing various pathogen-associated molecular patterns (PAMPs), either on cellular membrane, or in cytosol inducing a kind of selective autophagy that is called xenophagy (Deretic, 2011). Additionally, the autophagic machinery can be actively recruited upon phagocytosis of a pathogen *via* sensing and signaling by an extracellular PRR. In this context, a novel form of selective autophagy, termed LC3-associated phagocytosis (LAP) has been described in murine macrophages (Sanjuan et al., 2007). In LAP, autophagic protein LC3 is conjugated to the traditional, single-membrane, phagosome facilitating the induction of phagolysosome formation and maturation, and enhancing phagocytosis. This translocation is triggered upon TLR engagement by pathogens during phagocytosis and is dependent on common components of autophagic machinery such as PI3KC3–C1-associated proteins, ATG5 and ATG7 (Sanjuan et al., 2007; Martinez et al., 2015).

Apart from the elimination of phagocytosed pathogens, LAP is also associated with the uptake and clearance of apoptotic/necrotic dead cells or immune complexes by using phosphatidylserine or Fc receptors, respectively. Therefore, LAP pathway may protect from aberrant inflammatory responses and mediate immune tolerance (Martinez et al., 2011, 2016).

Although the vast majority of studies regarding autophagy as defense mechanism focus on macrophages, the first observation in neutrophils came from *in vitro* rickettsia-infected guinea pig peritoneal neutrophils, in 1984 (Rikihisa, 1984). Many years ago, the crucial *in vivo* role of autophagy in defense against pathogens has been demonstrated in mice knockout of autophagic factor *Atg5* in monocytes/macrophages, which have been found to be susceptible to infection with *Listeria monocytogenes* and *Toxoplasma gondii* (Zhao et al., 2008). Later on, an autophagy-independent *in vivo* role of *Atg5* in protection against experimental *Mycobacterium tuberculosis* by preventing neutrophil-mediated immunopathology in lungs has been suggested (Kimmey et al., 2015).

Evidence that autophagic machinery operates in human neutrophils was presented in 2010 (Mitroulis et al., 2010). Later on, transmission electron microscopic analysis of bacteria-containing autophagosomes and chemical inhibition of autophagy with 3-methyladenine (3-MA) or bafilomycin A1 (although non-specific) imply that xenophagy may play an antibacterial role in human neutrophils. (Itoh et al., 2015; Ramachandran et al., 2015; Rinchai et al., 2015).

Similar to macrophages, an interconnection between phagocytosis and autophagy pathway has been described in neutrophils. Phagocytosis of *Escherichia coli* triggers the autophagic machinery in neutrophils (Mitroulis et al., 2010). It has been also demonstrated that nicotinamide adenine dinucleotide phosphate (NADPH) oxidase activation and generation of ROS are required for the presence of LC3B in phagosomes of murine and human neutrophils (Huang et al., 2009; Mitroulis et al., 2010). Recently, it has been shown that human neutrophils undergo autophagy following *in vitro* infection with *Streptococcus pneumoniae* that depends on type III PI3K and ATG5 and enhances the rate of neutrophil phagocytosis of bacteria. ATG5 dependence was demonstrated by employing siRNA transfected neutrophils followed by incubation with granulocyte-macrophage colony-stimulating factor (GM-CSF) (Ullah et al., 2017).

Many pathogens have been shown to evade or exploit autophagy in macrophages, aiming to establish an intracellular niche for long-term survival and replication (Skendros and Mitroulis, 2012). Subversion of autophagy by microbes in neutrophils is far less studied. Previously, it has been demonstrated that adherent-invasive *Escherichia coli* strain (AIEC) isolated from Crohn’s disease patients can invade human neutrophils triggering the autophagic machinery. However, AIEC was able to escape killing by neutrophil-like PLB cells by disturbing autophagic flux at the autolysosomal step, which permitted intracellular survival of bacteria (Chargui et al., 2012).

Taken together, neutrophils probably use autophagy and phagocytosis both to kill pathogenic microbes and clear cellular debris, and a functional interrelationship between these two defense mechanisms could exist.

## Autophagy and Neutrophil Degranulation

Upon activation neutrophils release into phagosomes or secrete preformed antimicrobial and inflammatory proteins packed in cytoplasmic granules, in a process known as degranulation. There are four different types of granules in neutrophils: (a) primary or azurophilic granules (b) secondary or specific granules, (c) tertiary granules, and (d) secretory vesicles. Primary granules constitute the storage site of elastase, myeloperoxidase (MPO), cathepsins, and defensins, secondary granules contain mainly NADPH oxidase, lactoferrin, and matrix metalloprotease 9 (gelatinase), tertiary granules are enriched in gelatinase, but lack lactoferrin, while secretory vesicles are abundant in alkaline phosphatase and various cell membrane and plasma proteins derived from endocytosis (Cowland and Borregaard, 2016; Yin and Heit, 2018).

Notably, granule-derived proteins are required for the major neutrophil functions, including chemotaxis, antimicrobial function and NET release. Elastase and MPO do not only decorate NETs, but are also necessary for NET formation. Accordingly, various, highly concentrated, components of cytoplasmic granules are externalized at affected tissues *via* NET scaffold (Papayannopoulos et al., 2010; Metzler et al., 2011, 2014). Hence, degranulation and NET release are interconnected and share complementary roles during neutrophil activation.

The importance of autophagy in the regulation of neutrophil degranulation has been demonstrated in a study using myeloid-specific autophagy-deficient inflammatory mice models. Autophagy deficiency in neutrophils significantly reduced degranulation *in vitro* and *in vivo* (Bhattacharya et al., 2015). In the same study, ROS generation was also reduced in autophagy-deficient neutrophils, and inhibition of NADPH oxidase diminished neutrophil degranulation, suggesting that NADPH oxidase mediates the effects of autophagy on degranulation (Bhattacharya et al., 2015).

## Autophagy and Net Formation

In 2004, the group of A. Zychlinsky discovered a novel mechanism of neutrophil microbicidal activity, the release of NETs. NETs are a network of fibers that entraps and kills extracellular microbes (Brinkmann et al., 2004). NETs are extracellular chromatin strands carrying various highly active neutrophil-derived granular and cytosolic proteins. Notably, the effectiveness of neutrophil-derived mediators is significantly amplified due to their dense concentration in the fibrous network of NETs (Mitsios et al., 2016; Jorch and Kubes, 2017).

In contrast to apoptosis and necrosis, during NET-mediated cell death (NETosis) chromatin decondenses, the nuclear membrane disintegrates and the plasma membrane ruptures to release NETs (Remijsen et al., 2011a; Galluzzi et al., 2018). This process is also called suicidal NETosis, in contrast to vital NETosis, in which neutrophils are proposed to release NETs without losing their nuclear or plasma membrane, not undergoing cellular death (Pilsczek et al., 2010; Pieterse et al., 2016).

Besides the antimicrobial action of NETs, accumulating evidence highlighted their fundamental role in the pathogenesis of numerous non-infectious inflammatory disorders (Mitsios et al., 2016; Jorch and Kubes, 2017; Sollberger et al., 2018). Moreover, recent clinical and experimental studies suggest that in the context of different diseases, neutrophils release NETs that are qualitatively different and express disease-related bioactive proteins, determined by the disease inflammatory environment. For example, IL-1β-bearing NETs characterize inflammatory flares of typical autoinflammatory diseases such familial Mediterranean fever (FMF) and Still’s disease (Apostolidou et al., 2016; Skendros et al., 2017; Angelidou et al., 2018). Autoantigens in NETs have been associated with autoimmune diseases such as lupus, rheumatoid arthritis and ANCA-associated vasculitis (Lande et al., 2011; Khandpur et al., 2013; Tang et al., 2015; Lood et al., 2016), and exposure of thrombogenic tissue factor (TF) through NETs drives several thromboinflammatory conditions (Kambas et al., 2012a,b, 2014; von Brühl et al., 2012; Stakos et al., 2015; Chrysanthopoulou et al., 2017).

In order to better explain the variable protein load and action of NETs in different disorders, the “two-hit” model has been proposed. According to this, the inflammatory environment of each disease leads to transcriptional reprogramming in neutrophils inducing the expression of disease-related proteins (first-hit), and an additional stimulus (second-hit) enables NETs formation and extracellular exposure of these proteins via NETs (Mitsios et al., 2016; Skendros et al., 2017). One the other hand, NETs degradation by DNase I and phagocytic removal of NETs by macrophages, represent regulatory anti-inflammatory mechanisms aiming to balance excessive NETosis and limit tissue injury (Hakkim et al., 2010; Farrera and Fadeel, 2013).

**FIGURE 2:**
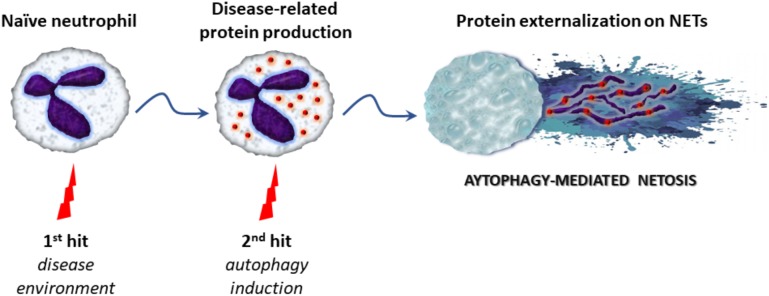
The proposed “two-hit” model in autophagy-mediated NETosis. Initially, various stimuli derived from the inflammatory microenvironment stimulate the production of disease-related proteins in naïve neutrophils (first-hit). Subsequently, triggering of autophagy (second-hit) leads to NET formation and the extracellular release of proteins *via* NET scaffold, contributing to antimicrobial capacity or inflammatory potential of the cell.

Over the last years, emerging evidence indicates that autophagy is tightly associated with NET formation, although the molecular mechanisms linking autophagy with NETosis are not clearly defined. Recent studies suggest that autophagy may represent the “second/NETotic-hit” (as mentioned above) leading to the extracellular delivery of NET-bound bioactive proteins (**Figure 2**; Stakos et al., 2015; Apostolidou et al., 2016; Skendros et al., 2017)

Neutrophil extracellular trap formation is triggered by many pathogenic agents and several proinflammatory stimuli, such as, cytokines (IL-8, TNFα), and interferon (IFN)α, whereas granular enzymes (MPO, elastase), and ROS positively regulate NET release (Mitsios et al., 2016; Papayannopoulos, 2018). Notably, there is a close interdependence between ROS production and autophagy, two major regulators of NETosis. ROS burst induce autophagy, which in turn is required to maintain efficient ROS production (Bhattacharya et al., 2015; Filomeni et al., 2015).

First, Remijsen et al. (2011b) showed that a combination of autophagy and ROS production is necessary for efficient PMA-induced-NET formation in human neutrophils. Inhibition of either autophagy or NADPH oxidase prevented the chromatin decondensation that is critical for NETosis, leading to apoptotic cell death. Furthermore, neutrophils isolated from patients with chronic granulomatous disease that lacking NADPH oxidase activity are incapable to generate NETs (Remijsen et al., 2011b). In parallel, our group demonstrated that neutrophils from patients with acute gouty arthritis exhibit autophagy-mediated spontaneous NET release, linking for the first time autophagy-associated NETosis with sterile inflammation (Mitroulis et al., 2011a). Subsequently, it has been indicated that mTOR and cytoskeletal machinery play a key role in regulating autophagy-mediated NET formation in human neutrophils. Pharmacological inhibition of the mTOR pathway significantly promoted autophagosome formation and histone citrullination facilitating NET release in response to *N*-formyl-methionyl-leucyl-phenylalanine (fMLP), whereas blockade of cytoskeletal dynamics abrogated mTOR/autophagy-mediated NETosis (Itakura and McCarty, 2013). Moreover, silencing of *ATG5* in AIEC-infected neutrophil-like PLB human cell line blocked NET formation (Chargui et al., 2012). Recently, it has also been shown that diminished expression levels of Atg5 contributed to reduced capacity of neutrophils to form NETs upon TLR2 ligand stimulation in aged mice, suggesting an important role of autophagy in maintaining the mechanism of NETs (Xu et al., 2017). Interesting, *in vitro* NET generation is impaired in older adults in response to LPS and IL-8 (Hazeldine et al., 2014), and reduced *ATG5* gene expression (Vieira da Silva Pellegrina et al., 2015; Xu et al., 2017). Consistent with this, inhibition of autophagy by pharmacological inhibitors or by small interfering RNA against *ATG7* attenuated LC3 autophagy formation and significantly decreased NET generation in promyelocytes (Ma et al., 2016). Furthermore, knockdown of the inhibitor of PI3K/AKT/mTOR pathway, autophagy inducer, PTEN in HL-60 differentiated neutrophil-like cells resulted in diminished generation of NETs upon stimulation with PMA (Teimourian and Moghanloo, 2015).

On the other hand, conflicting data have also been reported regarding the contribution of autophagy in NET release. In particular, *Atg5*-knockout mouse neutrophils, that exhibit reduced autophagic activity, preserved the capacity to release extracellular DNA. Furthermore, although PI3K inhibition prevented NET formation by human neutrophils, inhibition of late autophagy with bafilomycin A1 and chloroquine did not (Germic et al., 2017). This suggests that an autophagy-independent NETosis pathway may also exist (Pieterse et al., 2016; Germic et al., 2017).

### Autophagy/NET-Driven Response in Infection and Sterile Inflammation

Several studies have associated autophagy with the induction of NETs against various microbial agents *in vitro* and *in vivo* (Kenno et al., 2016; Sharma et al., 2017; Ullah et al., 2017). Autophagic machinery is also implicated in the induction of NETosis in experimental and human sepsis (Kambas et al., 2012a; Park et al., 2017). Interestingly, neutrophils isolated from non-surviving septic patients characterized by both impaired autophagy and decreased NET formation. Moreover, induction of autophagy protected mice from lethal sepsis in a NET-dependent fashion (Park et al., 2017).

Increasing evidence indicates the pathogenic role of autophagy-mediated NETs in various clinical models of acute or chronic sterile inflammation, including common IL-1β-mediated autoinflammatory diseases (Mitroulis et al., 2011a; Apostolidou et al., 2016; Papagoras et al., 2017; Skendros et al., 2017), ANCA-associated vasculitis (Kambas et al., 2014; Tang et al., 2015; Sha et al., 2016), active ulcerative colitis (Angelidou et al., 2018), severe asthma (Pham et al., 2017), cancer inflammation (Boone et al., 2015), and IL-17-mediated disorders such as fibrosis (Chrysanthopoulou et al., 2014) and epidermal hyperplasia (Suzuki et al., 2016).

The study of NETosis in IL-1β-mediated autoinflammatory diseases, such as FMF, provided novel mechanistic insights for the role of autophagy in the regulation of IL-1β inflammation (Apostolidou et al., 2016; Akdis and Ballas, 2017; Skendros et al., 2017). FMF is associated with mutations in the Mediterranean fever (MEFV) gene encoding protein pyrin, and is characterized by recurrent inflammatory attacks, often provoked by physical or psychological stress (Ozen et al., 2016).

Previously, it has been demonstrated that alterations in the levels of basal autophagy in neutrophils derived from FMF patients affect their inflammatory potential (Mitroulis et al., 2011b). It has been recently reported that induction of autophagic machinery is linked to the release of NETs-carrying IL-1β during FMF attacks, providing evidence for the involvement of neutrophil autophagy and NET formation in the regulation IL-1β-dependent response (Apostolidou et al., 2016).

Consisting with this, whole transcriptome analysis in neutrophils derived from FMF patients uncovered the role of autophagy-related protein regulated in development and DNA damage responses 1 (REDD1) as a key regulator linking environmental stress with autophagy-mediated NETosis and NET-associated IL-1β autoinflammation (Skendros et al., 2017). REDD1 is a key component of energy homeostasis and inflammation upregulated by various stressors such as glucocorticoids, adrenaline, DNA damage and hypoxia. It has been correlated with the regulation of autophagy through mTOR inactivation or oxidative stress (DeYoung et al., 2008; Qiao et al., 2015; Pastor et al., 2017). Apart from being a regulator of neutrophil-driven IL-1β response, it seems that also affects IL-1β maturation as demonstrated by the colocalization of REDD1 in autolysosomes containing pyrin and NALP3. MEFV mutations prevent localization of pyrin and NALP3 in REDD1 autolysosomes, enhancing IL-1β maturation and release through NETs (Skendros et al., 2017).

REDD1/mTOR/autophagy/NETosis pathway has been also associated with the IL-1β response in active ulcerative colitis supporting the autoinflammatory nature of this inflammatory bowel disease. Notably, in contrast to active Crohn’s disease, REDD1 or Beclin-1 expression in colonic neutrophils, and NETosis are diminished according to the distance from the inflamed intestinal area, suggesting that neutrophil autophagy could be a candidate diagnostic and disease severity target in ulcerative colitis (Angelidou et al., 2018).

Furthermore, recently it has been suggested that an unconventional secretory autophagy mechanism is also involved in the secretion of IL-1β by human neutrophils. Pharmaceutical inhibition of autophagy in primary neutrophils or knockdown of *ATG5* in neutrophil-differentiated PLB985 cells markedly reduced IL-1β secretion in culture supernatants after LPS and ATP stimulation. However, NET formation was not investigated in this study (Iula et al., 2018).

### Thromboinflammation

The concept of thromboinflammation or immunothrombosis, namely the dynamic cross-talk of innate inflammatory response with thrombosis is extensively studied in many experimental and clinical settings today (Gaertner and Massberg, 2016; Vazquez-Garza et al., 2017). In this context, autophagy emerges as a novel player linking proinflammatory NETs with the initiation and propagation of thrombosis.

In fact, autophagy was shown to mediate on NETs the delivery of functionally active TF, the main initiator of blood coagulation *in vivo*, arming neutrophils with potent thrombogenic capacity. This may be occurred either systemically, such as during the thrombophilic state that characterizes human sepsis, ANCA-associated vasculitis or severe ulcerative colitis (Kambas et al., 2012a, 2014; Angelidou et al., 2018), or locally at the affected coronary branch of myocardial infarction (Stakos et al., 2015; Chrysanthopoulou et al., 2017).

In a recent study utilizing *ex vivo* human system and i*n vivo* mice model of arterial thrombosis, it has been indicated that activated platelets of acute ST-segment elevation myocardial infarction (STEMI) patients release inorganic polyphosphate (polyP) in a thrombin-dependent manner, which subsequently induce NET formation in TF-expressed neutrophils. This mechanism is fine-tuned by autophagy involving the phosphorylation status of mTOR. Importantly, antiviral interferon IFN-λ1/IL-29 emerged as a novel naturally occurring agent that exerts a strong inhibitory effect on NET formation by balancing the action of polyP on mTOR/autophagy pathway (Chrysanthopoulou et al., 2017).

Additionally, an important role for platelet-exposed high mobility group box 1 (HMGB1) in activating autophagy-mediated NET generation has been suggested in a study using thrombi biopsies from acute myocardial infarction patients, pharmacologic and genetic tools (Maugeri et al., 2014).

Taken together, neutrophil autophagy is proposed as a central rheostat of NET-driven tromboinflammation in acute coronary syndrome, and probably other related thrombotic conditions.

## Targeting Autophagy in Infections and Neutrophil-Mediated Diseases

**FIGURE 3:**
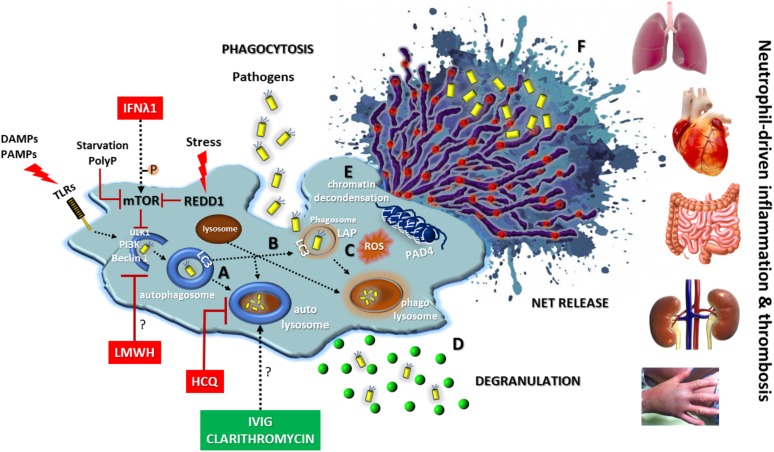
Autophagy in neutrophil biology and disease. Autophagy has a central key role in neutrophil biology by **(A)** promoting intracellular elimination of pathogens (xenophagy), **(B)** accelerating phagocytosis (LAP), **(C)** interplaying with ROS, **(D)** regulating degranulation, **(E)** facilitating the resolution of inflammation *via* NETotic cell death, and **(F)** leading to externalization of various NET-bound bioactive proteins (depicted as red spherical spots). The enhancement of autophagic machinery (e.g., IVIG, clarithromycin) increases the antimicrobial capacity of neutrophils. On the contrary, blocking autophagy pathway at initial (e.g., IFNλ1, LMWH) or late steps (e.g., HCQ) could be beneficial for neutrophil-driven inflammatory or thrombotic diseases. REDD1/mTOR pathway is a main regulator of autophagy in neutrophils. DAMPS, damage-associated molecular patterns; PAMPS, pathogen-associated molecular patterns; P, phosphorylation; PolyP, inorganic polyphosphate; REDD1, regulated in development and DNA damage responses 1; LAP, LC3-associated phagocytosis; ROS, reactive oxygen species; HCQ, hydroxychloroquine; LMWH, low molecular weight heparin; IVIG, intravenous immunoglobulin; Symbol “?” denotes the possible target of the anti-autophagic action.

The above described key role of autophagy in neutrophil biology denotes that elements of autophagic machinery could be effective therapeutic targets for the enhancement of antimicrobial defense or the amelioration of neutrophil/NET-driven inflammation and thrombosis, either as a monotherapy or in combination with classical regimens (**Figure 3**). Combining drugs that act on different targets within the regulatory network of autophagy could be more efficacious than one drug (Iyengar, 2013).

According to the above, intravenous immunoglobulin (IVIG) preparations enhanced *in vitro* both the bactericidal activity and phagocytosis-mediated autophagy of neutrophils isolated from healthy donors, as well as from immunocompromised patients after HSC transplantation, against multidrug-resistant or drug sensitive *Escherichia coli* and *Pseudomonas aeruginosa* strains (Itoh et al., 2015; Matsuo et al., 2015). In another human study, macrolide antibiotic clarithromycin was found to induce *ex vivo* and *in vitro* the release of NETs decorated with the potent antimicrobial peptide cathelicidin LL-37. LL-37-bearing NETs exhibited strong *in vitro* inhibitory activity against multi-drug resistant *Acinetobacter baumannii* growth and biofilm formation (Konstantinidis et al., 2016). Together, these observations imply that targeting autophagy-promoted NETs may present a novel therapeutic strategy to improve infection defense in the aged or immunocompromised individuals.

On the other hand, blocking autophagy might be beneficial in several devastating neutrophil-mediated inflammatory diseases. For example, hydroxychloroquine (HCQ), an old, low-toxicity and low-cost anti-rheumatic drug, is an inhibitor of autophagy impairing both the autophagosome-lysosome fusion and the degradation of the autophagosome contents (Rockel and Kapoor, 2016). In accordance with this, HCQ administration was associated with inhibition of autophagy-mediated NET release preventing disease relapses and reducing the needs for glucocorticoids and anti-IL-1 agents in a case of difficult-to-treat adult-onset Still’s disease (Papagoras et al., 2017). In addition, HCQ is a mainstay treatment in systemic lupus erythematosus, a well-defined NET-mediated autoimmune disease (Bosch, 2011; Rockel and Kapoor, 2016).

Recently, it has been reported a novel, autophagy-based, anti-inflammatory action of low molecular weight heparins (LMWH) in peripheral blood neutrophils. Treatment of healthy volunteers with prophylactic doses of LMWHs hindered the ability of neutrophils to activate autophagy and to generate NETs in response to inflammatory stimuli, such as IL-8, PMA, and HMGB1 (Manfredi et al., 2017).

Experimental evidence also suggests that repositioning of IFN-λ1/IL-29 may provide a novel anti-autophagic therapeutic strategy against thromboinflammation that do not interfere with normal hemostasis (Chrysanthopoulou et al., 2017).

## Conclusion

Autophagy is a key mechanism that is implicated in quite all aspects of neutrophil biology and pathophysiology. The balance of autophagic response in neutrophils is critical for cellular homeostasis and host health. According to environmental danger, autophagy behaves as a double-edged sword for the host neutrophils. It is beneficial by fighting various pathogens and preventing their growth and chronic parasitism. Instead, it is harmful by inducing potent inflammatory responses, including NET formation on systemic and tissue-level. This encourages the design of novel therapeutic agents and/or the repositioning of old drugs targeting autophagic machinery in diseases with crucial involvement of neutrophils/NETs in their pathogenesis. To this end, analysis of big data provided by system biology approaches are urgently needed today.

## Author Contributions

PS and IM wrote the manuscript and created the figures. KR critically reviewed the manuscript.

## Conflict of Interest Statement

The authors declare that the research was conducted in the absence of any commercial or financial relationships that could be construed as a potential conflict of interest.
